# Prevalence of anxiety, depression, stress, and perceived stress and their relation with resilience during the COVID‐19 pandemic, a cross‐sectional study

**DOI:** 10.1002/hsr2.460

**Published:** 2022-01-06

**Authors:** Seyedeh Yasamin Parvar, Narges Ghamari, Fatemehsadat Pezeshkian, Reza Shahriarirad

**Affiliations:** ^1^ Student Research Committee Shiraz University of Medical Sciences Shiraz Iran; ^2^ Bone and Joint Diseases Research Center Shiraz University of Medical Sciences Shiraz Iran; ^3^ Thoracic and Vascular Surgery Research Center Shiraz University of Medical Sciences Shiraz Iran

**Keywords:** anxiety, COVID‐19, depression, mental health, resilience, stress

## Abstract

**Background and aims:**

Home quarantine and physical distancing at the time of coronavirus disease 2019 (COVID‐19) had a severe effect on the mental health of the populations. Resilience has been reported previously to be a protective factor against anxiety, stress, and depression. This study evaluates the prevalence and severity of depression, anxiety, stress, and perceived stress and their relation with resilience associated with the COVID‐19 pandemic among a sample of the general population in Southern Iran.

**Methods:**

In this cross‐sectional web‐based survey, from April 12 to May 13, 2020, stress, anxiety, depression, perceived stress, and resilience were measured using the Persian version of Depression Anxiety and Stress Scale (DASS‐21), Perceived Stress Scale (PSS‐14), and Connor‐Davidson Resilience Scale (CD‐RISC). Statistical analyses were carried out using the IBM Statistical Package for Social Sciences. Mean ± standard deviation (SD) and frequencies were used to describe demographic data. Independent sample *t*‐test, Spearman correlation, and the Pearson correlation coefficient were performed to examine anxiety, depression, stress, and resilience.

**Results:**

Among a total of 538 participants, the overall prevalence of moderate‐to‐extremely severe depression, anxiety, and stress was found to be 26.1%, 33.2%, and 5.8%, respectively. The overall median PSS and resilience score were 30 and 70, respectively. There was a significant association between higher age and perceived stress. Male and high income were related to higher resilience scores. Perceived stress positively correlates with resilience, whereas depression significantly correlates with anxiety and stress. Individuals with underlying disease demonstrated significantly higher scores for depression and anxiety. Also, perceived stress had a significant but weak, positive correlation with age and the number of quarantine days.

**Conclusion:**

The occurring COVID‐19 pandemic could be the culprit of psychological distress, anxiety, and depression of large population quantities. Our results showed a subordinate overall resilience in the general Iranian population during the COVID‐19 pandemic.

## 1 INTRODUCTION

On March 11, 2019, the World Health Organization reported the coronavirus disease 2019 (COVID‐19) as a global pandemic, forcing many countries to adopt limiting policies that they have never carried out before. In addition to threatening human health, pandemic diseases can also trigger irreversible psychological effects globally.^1^, ^2^, ^3^ Therefore, the mental health and psychosocial life during the COVID‐19 pandemic should be considered as much as the clinical aspects.^4^, ^5^ Several studies in many countries have supported the mental impact of the COVID‐19 pandemic on the populations' everyday lives.^5^, ^6^ Approximately 21% of individuals worldwide faced clinically significant depression, and between 6% and 51% of people have reported anxiety symptoms during the COVID‐19 pandemic, which is higher than the previously reported rates before the pandemic.^6^, ^7^ Pakistan, Thailand, the Philippines, and Malaysia had the highest rates of depression, anxiety, and stress among middle‐income countries.^8^ At the time of our study, Iran was the second‐highest country in Asia in COVID‐19 cases^9^, ^10^ and had the highest anxiety score (mean = 7.83%) among eight countries and three continents.^11^ Since then, the high mortality rate among the Iranian population and the new adapted protocols caused a new era unlike before.^9^

Quarantine and physical distancing have led to severe stress and depression among healthcare personnel, frontline groups, and other population members.^12^ Contrary to these results, depressive symptoms were significantly lower in countries with strict government policies. Thereby, facemasks and lockdown have been reported to have a protective effect on mental health during the COVID‐19 pandemic in another study.^7^, ^13^ According to the study on the Vietnamese population evaluating the effect of lockdown on people's mental health, the reported prevalence of stress, anxiety, and depression was less than 10%, which was significantly lower than the previously reported studies in Iran and Italy.^14^ According to the survey in the north of Iran, more than 70% of the COVID‐19 patients experienced depression, anxiety, and perceived stress.^15^ Moreover, a large sample study among the Chinese population has also reported that about a third of participants developed psychological distress during the COVID‐19 pandemic.^16^ These factors and the massive social media news on the COVID‐19 outbreak affected the population's mental health and copping‐appraisal process with unknown long‐term psychological outcomes. This impact was more prominent among patients with preexisting mood disorders who are at increased risk of hospitalization and mortality.^4^, ^11^, ^17^, ^18^, ^19^

Resilience is the process of successfully adapting to life adversities and situations, including trauma, tragedy, danger, and high amounts of stress. It is a dynamic, complex, and multidimensional manner with varying degrees in different patients.^20^ Previous reports have highlighted the significant negative association between resilience and mental health and reported resilience as a protective factor against anxiety, stress, and depression.^21^, ^22^ Furthermore, a higher resilience level and copying strategies during the COVID‐19 pandemic were reported to be associated with lower rate of suicidal ideation, which is one of the most severe mental health impacts of this pandemic. Therefore, it is necessary to study COVID‐19 psychological consequences as individuals with anxiety and depression were found to have significantly higher suicidal ideation and lower resilience.^23^, ^24^ It is also worth mentioning that there is a significant correlation between childhood traumatic experiences such as this pandemic and sensory processing patterns. The level of individuals’ sensory processing patterns is a critical factor in determining patients' quality of life and clinical outcomes.^25^

In this regard, Connor‐Davidson Resilience Scale (CD‐RISC) was developed as a reliable measure of reporting psychological resilience and has been validated in several populations. It has high internal continency, and it is a potent predictor of mental health related quality of life; thereby, the CD‐RISC can be used in the clinical context.^26^ Connor and Davidson corresponded the 25 items of this questionnaire to five factors, including having high standards and competence, handling negative emotions and perceived benefits of stress, secure relationships and having positive attitudes toward changes, perceived control, and spirituality.^27^

Based on the persisting high mortality rates and an unknown timeline until effective treatment and vaccination have been assured, evaluating factors relating to the general population's mental health is of high value. Therefore, the present study aims to assess the effect of resilience on depression, anxiety, stress, and perceived stress among a sample of the general population and the prevalence and severity of each disorder in Iran and based. We hypothesized that resilience would significantly predict psychological symptoms, mainly stress, anxiety, and depression. Our study also aimed to investigate the possible effect of socio‐demographic features on the mentioned factors.

## 2 METHODS

### 2.1 Study design and participant recruitment

This cross‐sectional web‐based survey was conducted among a sample of the general population in southern Iran. Estimation of the sample size was based on a study by Verma et al^28^ by considering 28% frequency of anxiety as one of the main factors and based on n = Z 2 *P (1 − P)/d2 with a confidence level = 95%, power of 28%, and d (margin of error) = 0.15, reaching a calculated sample size of 440 participants.

The questionnaire was anonymously distributed throughout various multimedia platforms and the correspondence email address in case of any issues about the project. A snowball sampling method, concentrated on recruiting the general public living in southern Iran during the first wave of COVID‐19 pandemic, was utilized. The survey was first distributed to university students and healthcare personnel, and they were encouraged to pass the provided link to the different groups of people across various online platforms (eg, WhatsApp, Instagram, student blogs, etc). To avoid the potential risk of selection bias, the provided link has been sent several times in several platforms and by the help of a large group of people. Volunteers to participate could enter the study by selecting a provided link, which would redirect them to our questionnaire. A brief report of the importance of the scales was presented on the questionnaire's cover page, and 71 multiple choice questions followed after that. It was also stated that the questionnaire was noncommercial and voluntary and took about 14 minutes to complete. Furthermore, IP filtering was used to prevent multiple answers and duplicates from one system.

### 2.2 Eligibility criteria

Inclusion criteria consist of all literate participants age between 7 and 18 years with their guardian or parents' consent and age 18 years and above with their own approval of online inform consent, living in Iran since the first case of COVID‐19 was detected at February 19, 2020, and have access to the web‐based platform. Non‐Persian speakers, residents of others countries, and diagnosed mental disorder were excluded from the study through a first page questions. There were no other inclusion/exclusion criteria since we decided to include various groups of people to assess the relationship between different epidemiologic, social, and economic features and the mental health of the populations during the pandemic.

### 2.3 Data collection and outcome assessment

**FIGURE 1:**
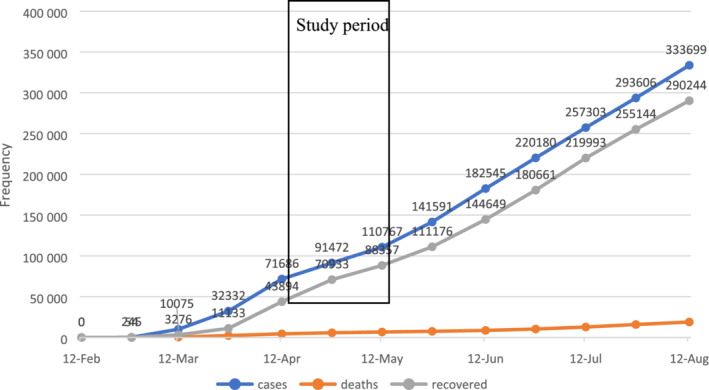
Report of coronavirus disease cases, deaths, and recoveries in Iran during 2020

The questionnaire was distributed among 1611 participants from April 12 to May 13, 2020. Data collection was initiated when Iran reported 71 686 cases of infection and 4474 deaths attributed to COVID‐19 (Figure 1).

Participants filled out necessary demographic information, including age, gender, marital status, employment status, educational level, income level, underlying diseases, and residency city. They were further asked to answer related questions about the COVID‐19 pandemic, including the entire quarantine days and history of positive COVID‐19 PCR in themselves or other family members. To assess the psychological dimension of the COVID‐19 and mental health status of participants, three well‐developed and valid questionnaires, the Persian version of Perceived Stress Scale‐14 (PSS‐14), the 21 item Depression Anxiety and Stress Scale (DASS‐21), and Connor‐Davidson Resilience Scale (CD‐RISC), were administrated to all participants. The high reliability of the Persian version of these three questionnaires was reported in Iranian population^29^, ^30^, ^31^, ^32^ The participants who had not agreed to share their answers and patients with incomplete and repetitive questionnaires were excluded from study.

#### 2.3.1 Perceived stress scale‐14 item

The PSS is the most widely used tools for assessing clinical and nonclinical stress. It is a self‐reported instrument showing participants' self‐awareness of stress. PSS has been designed in 1983 by Cohen et al^33^ and comes in three versions, including the 4‐, 10‐, and 14‐item questionnaire. We used the 14‐item questionnaire for our study, which has a satisfactory internal consistency.^34^ This scale aims to assess the thoughts and feelings about stressful events, controlling, copping, and overcoming stress that occurred within the last month. Questions included “In the last month, how often have you felt nervous and ‘stressed’?” “How often have you found that you could not cope with all the things that you had to do?” “How often have you been able to control irritations in your life?” “How often have you felt that you were on top of things?” and other similar questions. Scores were based on the Likert scale, consisting of 0 as never, one as sometimes, two as half of the time, three as most of the time, and four as always. The total scores ranged from 0 to 56, while we assigned a cut‐off point of 28, assuming scores equal or greater than 28 as greater stress‐related to COVID‐19. The Cronbach's alpha coefficients for this questionnaire ranged from 0.84 to 0.90 in previous studies.^35^

#### 2.3.2 Depression anxiety and stress scale‐21 item

DASS is another self‐report questionnaire for assessing participants' mental health, made up of 21 items including three emotional states: Depression, anxiety, and stress, in which the Cronbach's alpha coefficients for three subscales were estimated as 0.93, 0.88, and 0.82, respectively, in a large sample in England.^36^ DASS‐21 has been used in a study conducted in Iran by Ashghari et al and has shown high internal consistency, including 0.93, 0.90, and 0.92 and high test–retest reliability over 3 weeks, including 0.84, 0.89, and 0.90 for depression, anxiety, and stress, respectively. Moreover, the intraclass correlation confidence between two executions was reported 0.78, 0.87, and 0.80 for depression, anxiety, and stress, respectively.^37^ DASS‐21 has been validated in assessing mental health in several countries during COVID‐19 pandemic, including Iran,^38^ Philippines,^39^ Poland,^13^ the United States,^40^ Spain,^41^ and China.^42^

The DASS‐21 scale used in this study is a short form of the original 42‐item version created in 1995.^43^ Therefore, the scores on each of the three subscales were summed and multiplied by 2, including 7 items in each subscale based on a 4‐point Likert scale from 0 indicating “Does not apply to me at all” or “never” to 3 indicating “very much applies to me” or “always.” The total score ranges from 0 to 120. Table 1 shows each subscales scoring system.

**TABLE 1:** The DASS‐21 subscales scoring

	Depression	Anxiety	Stress
Subclinical	0‐9	0‐7	0‐14
Mild	10‐13	8‐9	15‐18
Moderate	14‐20	10‐14	19‐25
Severe	21‐27	15‐19	26‐33
Extremely severe	≥28	≥20	≥34

Abbreviation: DASS‐21, depression, anxiety, stress scale 21.

#### 2.3.3 Connor–Davidson resilience scale

This is a 25‐item questionnaire created by Connor and Davidson in 2003.^27^ They believe that this scale can highly estimate resilience degree in various populations. This scale's psychometric aspects have been studies in six groups, including the general population, referrals to primary care units, psychiatrists' outpatients, generalized anxiety disorder, and two groups of posttraumatic stress disorder patients.^44^ The scoring system was based on a 5‐point Likert scale from 0 (not true at all) to 4 (true nearly all the time). Statements such as “I am able to adapt when changes occur,” “I have one close and secure relationship,” “Past successes give me confidence,” “I can deal with whatever comes my way,” and “Sometimes fate or God helps me” are presented in this questionnaire. The total point of CD‐RISC is calculated by summing up all questions scores, computing a range from 0 to 100. We assigned a cut‐off point of 50 for our study, assuming higher scores indicate greater resilience. The Cronbach's alpha coefficients of the questionnaire have been reported 0.89, while its test–retest reliability over 4 weeks has been reported to be 0.87 in previous studies.^44^ The Cronbach's alpha coefficients for the Persian version of CD‐RISC were reported as 0.89 by Mohammadi et al.^45^ This version includes five major subscales: The first factor reflects “having high competence and standards” (eight items), the second one is “handling negative emotions and trusting one's instincts” (seven items), the third factor is “able to adapt to change and secure relationships” (five items), the fourth factor reflects “perceived control” (three items), and the fifth factor is “spirituality” (two items).

### 2.4 Statistics

Statistical analyses were carried out using the IBM Statistical Package for Social Sciences (SPSS, version 18; SPSS Inc., Chicago, Illinois). Mean ± standard deviation (SD) and frequencies have been used to describe the demographic data. The median and interquartile rates [IQR] have been used to describe the PSS‐14, DASS‐21, and CD‐RISC scores according to each demographic factor. Proportions and percentages are accompanied by the actual numerator. Analysis of variance (ANOVA) or independent sample *t*‐test and Spearman correlation were also performed to examine the correlation between anxiety, depression, stress, and resilience. The Pearson correlation coefficient was performed to measure the linear correlation between dependent variables, including depression, anxiety, stress, perceived stress, age, and quarantine days. The *r* between 0.7 and 1 indicates a strong correlation between the variables, whereas if the *r* is between 0 and 0.3, it indicates a weak correlation. Finally, the significance level of the *P*‐value was set at .05.

### 2.5 Ethical considerations

The present study was approved by the Medical Ethics Committee of Shiraz University of Medical Sciences according to the declaration of Helsinki. Our study's aim was completely explained to the participants, and they were assured that their information would be kept confidential by the researcher. The participants agreed to participate in the study by reviewing the questionnaire's cover page and clicking on the provided link. Participants younger than the age of 18 were asked to answer the questions with a parent or guardian's assistance.

## 3 RESULTS

From a total of 1061 people who viewed our online survey, after excluding incomplete answers, 538 (50%) of the individuals filled out the form completely, included in the study, and were analyzed from April 12 to May 13, 2020. Table 2 summarizes the descriptive information on socio‐demographic features of the study population. The mean age of the population was 38.0 ± 10.5 years (range: 13‐72), and 74.2% (n: 399) were female. Also, 384 (71.4%) were married, and 339 (66.7%) had a low‐income salary. Among our population, 401 (74.5%) had a university degree including associate degree (35: 8.7%), bachelor (204: 50.9%), master (118: 29.4%), and PhD (44: 11.0%). Also, 313 (58.3%) were employed at the time of our survey, including 48 (15.3%) students, 51 (16.3%) health care workers, 113 (36.1%) governments' employee, 9 (2.9%) in private offices, 19 (6.1%) teachers, and 73 (23.32%) participants had other jobs. Furthermore, 43 (8%) reported having an underlying disease. The mean quarantine days of the study population were 42.4 ± 19.6 days (range 0‐120). Seven case out of 538 individuals (1.3%) that participated in the program had positive COVID‐19 test themselves or among their close family members.

**TABLE 2:** The median and interquartile range for PSS, DASS‐21, and CD‐RISC based on each demographic variable in a sample of the general population in Southern Iran

Variable	Frequency (%)	PSS	*P*‐value^a^	Depression	*P*‐value^a^	Anxiety	*P*‐value^a^	Stress	*P*‐value^a^	CD‐RISC	*P*‐value^a^
Age											
≤30	129 (24.0)	29 [26‐32]	.19	10 [8‐14]	.40	6 [2‐12]	.43	6 [2‐10]	.49	67 [53‐81]	.42
31‐45	290 (53.9)	30 [27.7‐33]		10 [1.5‐12.5]		6 [1.5‐12]		4 [1.5‐8]		70 [80‐52.7]	
>45	119 (22.1)	30 [28‐33]		10 [0‐14]		6 [0‐12.5]		4 [0‐12]		71 [56‐82]	
Gender											
Female	399 (74.2)	30 [28‐33]	.10	10 [2‐14]	.34	6 [2‐12]	.43	4 [2‐8.5]	.27	69 [53‐79]	.01
Male	139 (25.8)	30 [26‐33]		10 [2‐14]		6 [2‐12]		4 [2‐10]		72 [83‐57]	
Marital status											
Married	384 (71.4)	30 [27‐33]	.25	10 [2‐14]	.58	6 [2‐12]	.78	4 [2‐10]	.99	70.5 [55‐82]	.07
Unmarried	154 (28.6)	30 [26‐32]		10 [3.5‐14]		6 [2‐12]		4 [2‐10]		67 [53‐78]	
Income											
Low	339 (66.7)	30 [27‐33]	.37	10 [2‐14]	.46	6 [2‐12]	.70	4 [2‐10]	.58	68 [52‐77]	<.001
High	199 (33.3)	30 [28‐33]		10 [2‐14]		6 [2‐12]		4 [2‐8]		73 [59‐86]	
Education											
University degree	401 (74.5)	30 [26‐33]	.19	10 [2‐12]	.48	5 [2‐10]	.15	4 [2‐8]	.18	69 [48‐82.5]	.26
Diploma and under diploma	137 (25.5)	30 [27‐33]		10 [2‐14]		6 [2‐12]		4 [2‐10]		70 [55‐80]	
Employment status											
Employed	313 (58.3)	30 [27‐32]	.51	10 [2‐14]	.80	6 [2‐12]	.91	4 [2‐9]	.68	70 [55.5‐81.5]	.08
Unemployed	224 (41.7)	30 [27‐33]		10 [2‐14]		6 [2‐12]		4 [2‐10]		69 [52‐87.7]	
Underlying disease											
Negative	495 (92.0)	30 [27‐33]	.06	10 [2‐14]	.03	6 [2‐12]	.02	4 [2‐10]	.091	70 [54‐80]	.42
Positive	43 (8.0)	29 [26‐31]		10 [8‐14]		8 [2‐12]		4 [2‐12]		68 [50‐82]	
COVID‐19 PCR^b^											
Negative	531 (98.7)	30 [27‐33]	.97	10 [2‐14]	.32	6 [2‐12]	.09	4 [2‐10]	.16	70 [54‐81]	.68
Positive	7 (1.3)	30 [27‐32]		4 [0‐14.5]		1 [0‐6]		1 [0‐7]		68 [40‐79]	

*Note*: Data are described as frequency (percentage) or median [interquartile range].

Abbreviations: CD‐RISC, Connor‐Davidson resilience scale; DASS‐21: depression anxiety stress scale 21; PSS, perceived stress scale.

^a^ Mann–Whitney *U* or Kruskal–Wallis *H* test.

^b^ Positive test for coronavirus disease 2019, polymerase chain reaction, in themselves or family members.

**FIGURE 2:**
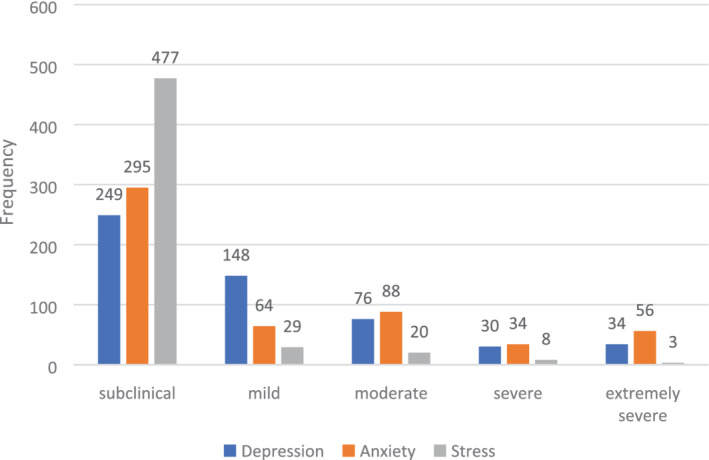
Frequency of subclinical to extremely severe depression, anxiety, and stress among the population based on the depression, anxiety, stress scale 21 (DASS‐21) questionnaire (N: 538)

Depression, anxiety, and stress were evaluated among the population of our study, in which Figure 2 displays the frequency of participants in each of the five categories of DASS‐21 based on subclinical, mild, moderate, severe, and extremely severe. The overall prevalence of moderate‐to‐extremely severe, also known as clinically significant, depression, anxiety, and stress among participants were 26.1%, 33.2%, and 5.8%, respectively.

The overall PSS score among the study population was 30 [IQR = 27‐75]. The median score for depression, anxiety, and stress subscales was 10 [2‐14], 6 [2‐12], and 4 [2‐10], respectively. Based on the Mann–Whitney *U* analysis, individuals with underlying disease demonstrated significantly higher scores for depression and anxiety (*P*‐value = .03 and .02, respectively). Furthermore, the resilience score was significantly lower in participants with lower income (*P* < .001) and also in females (*P*‐value = .01).

The scores for each subgroup based on demographic characteristics are shown in Table 2. The overall score of CD‐RISC among the study population was 70 [IQR 54‐81]. According to the cut‐off point of 50, a total number of 438 individuals (81.4%) scored over 50 and, therefore, had high levels of resilience.

The correlation among DASS‐21, CD‐RISC, and PSS scores, along with age and number of quarantine days, was evaluated based on the Spearman correlation analysis (Table 3). As demonstrated, perceived stress had a significantly weak positive correlation with resilience (*P*‐value = .01, *r* = 0.106), whereas depression had a significantly strong positive correlation with both anxiety and stress (*P* < .001, *r* = 911 and *P* < .001, *r* = 0.9, respectively). Anxiety also had a significantly strong positive correlation with stress (*P* < .001, *r* = 0.935). Also, perceived stress had a significant, but weak, positive correlation with age and number of quarantine days (*P*‐value = .04, *r* < 0.091 and *P*‐value = .002, *r* = 0.134, respectively).

**TABLE 3:** The Spearman correlation among perceived stress, depression, anxiety, stress, and resilience toward coronavirus disease 2019 (COVID‐19) among the general population

Variables	Analysis	Resilience	Depression	Anxiety	Stress	Perceived stress	Age
Depression	Correlation coefficient	−0.005	1				
	Sig (two‐tailed)	.91	‐				
Anxiety	Correlation coefficient	−0.011	0.911^a^	1			
	Sig (two‐tailed)	.79	<.001	‐			
Stress	Correlation coefficient	−0.012	0.900^a^	0.935^a^	1		
	Sig (two‐tailed)	.78	<.001	<.001			
Perceived stress	Correlation coefficient	0.106^b^	−0.050	−0.053	−0.043	1	
	Sig (two‐tailed)	.01	.24	.22	.32	‐	
Age	Correlation coefficient	0.082	−0.017	0.003	0.025	0.091^b^	1
	Sig (two‐tailed)	.06	.70	.95	.56	.04	‐
Quarantine days	Correlation coefficient	0.002	−0.003	0.021	0.009	0.134^b^	−0.013
	Sig (two‐tailed)	.95	.95	.63	.84	.002	.76

^a^ Correlation is significant at the .01 level.

^b^ Correlation is significant at the .05 level.

## 4 DISCUSSION

This study showed that the overall prevalence of moderate‐to‐extremely severe depression, anxiety, and stress was 26.1%, 33.2%, and 5.8%, respectively. Noticeable association between the perceived stress and the higher age was observed. Furthermore, the male gender and high‐income family were protective factors for maintaining a good resilience. Individuals carrying an underlying disease scored significantly higher for anxiety and depression. Lastly, perceived stress had a weak but yet noticeable, positive relation with the number of quarantine days and the age. As these results show, the COVID‐19 mental impact is an important issue that should be addressed.

The COVID‐19 pandemic brought unique challenges to the general public. These challenges are complex and include the short‐ and long‐term physical and mental health impacts; change in work and family lifestyles; the possibility of infection; changes in dynamics of household and relationships; anxiety about the risks of infection; and coping and dysregulation of mood. The pandemic is also accompanied by an enormous storm of stress, involving acute crisis and loss and a high potential risk threatening mental health and resilience.^5^, ^46^ Resilience as a protective power against mental instability proves to be a significant factor worth investigating.

Our study was conducted during the early periods of the pandemic in Iran, in which the general population had just been exposed to this new phenomenon and had little knowledge and experience with the newly imposed situations.^47^ The overall score of CD‐RISC among the general Iranian population in our study was 70, while in a similar study, Killgore et al^48^ reported a resilience score of 66.84 based on CD‐RISC among the US participants during the COVID‐19 lockdown. Compared to published normative data for this scale, Iranian and US general populations demonstrated significantly lower resilience levels during the COVID‐19 pandemic lockdown. These data suggested that the self‐perceived psychological resilience among both Iranian and US inhabitance could have been adversely influenced due to the ongoing crisis, probably through acute changes in emotional perception or perceived support. Their study also reported that lower resilience was correlated with the fear of COVID‐19 permanency.^48^ There is a close relationship between resilience and mental well‐being, in which a lack of resilience as a resource for effective coping can suggest a need for psychosocial support during somatic disease care in clinical practice and government policies should be focused on addressing these issues due to the vulnerability of our society.^49^

Gender was among the main factors evaluated in our study, in which male participants had a significantly higher resilience than female participants at the same time that stress perception was not significantly different between the genders. However, regardless of the occurring pandemic, female gender was always perceived to make up the majority of depression prevalence in different communities.^50^ This result was in line with another study that puts healthcare workers under investigation and found higher resilience men, respectively.^51^ Predominantly, male individuals have higher resilience than women. Hirani et al concluded that this discovery could be because the concept of resilience does not reflect how gender role, social expectations, perception, and environmental factors interact to differentially form women's and men's experiences and the way they acknowledge adversity.^52^ Limura et al claimed that women significantly display higher levels of conscientiousness than men. Conscientiousness is thought to be vital to maintain positive interpersonal relationships, and thus it could be more evolutionally emphasized in women than in men.^53^ Furthermore, Barzilay et al noted a higher rate of worries regarding COVID‐19 in the women under study than men.^54^ This finding gives strength to our result as to why the women bear more stress and, therefore, less resilience. A study carried out in a sample of people in China in the early days of the pandemic, similar to the period of our study, regarded the female gender as a risk factor for psychological distress.^16^ Analogously, Italian studies are also in line with this finding by highlighting the role of gender in predicting distress regarding COVID‐19.^55^ Beneria et al also concluded that in those who work in a health care unit, women had scored higher perception of stress in the PSS‐14 than men.^56^ Our study also showed that persevered stress and resilience were in direct correlation with each other; Southwick et al reported with potential psychological, social, spiritual, and neurobiological approaches to enhancing stress resilience, decreasing the likelihood of developing stress‐induced depression/anxiety, and treating stress‐induced psychopathology in achievable.^57^

Another discovery that we were able to achieve was that gender did not appear to have a consequential effect on the DASS‐21 score of depression, anxiety, and stress. Contrary to our results, being female was associated with higher degrees of psychological distress in Vietnamese population,^58^ and Chinese male adults were reported to have a significantly higher severity of depression than female participants.^59^ However, a Philippian study conflicts with the Chinese results in ways that it deduced that the female gender was significantly associated with the a greater psychological impact of the pandemic and higher levels of stress, anxiety, and depression.^39^ This finding appears to be in correlation with another study, discovering the significant difference in depression, anxiety, and stress across gender.^60^ Previous studies appear to be in line with results, which have unvaryingly come across correlations between female genders and raised psychological disturbances.^16^, ^55^, ^61^ This could be elucidated by the fact that women cope with stressful circumstances through expressing their feeling and, as a result, are more liable to complaints about psychological and physical symptoms.^62^, ^63^ Following Wang et al, anxiety disorders are inspected at threefold higher levels in Chinese women than in men during the pandemic.^61^ Eventually, the female gender could be identified as the most influential predictor of posttraumatic stress disorder symptoms after pandemics.^64^

Regarding income, those with higher income had significantly higher resilience than those with low salaries. This is correlated with what was expected to see, as higher‐income brings a sense of security in one's life. COVID‐19 pandemic had a great impact on many occupations, which led to the shutdown of the financial market and introduced a new concern globally. Ozili et al stated a positive correlation between the increasing number of lockdown days and the level of economic activities.^65^ All this turns into a potential threat to the future of those with low income and lowers their tolerance regarding the COVID‐19 situation.

In our study, age was found to have a noticeable direct relation with stress perception, in a manner that higher age was associated with higher perceived stress. Higher age and life experiences bring a higher perception of community and, as a result, higher intake of possible risks threatening each individual's quality of life. Demetriou et al also concluded that resilience has a positive relation with age; as age advances, resilience appears to increase.^66^ Contrary to our results, Klein et al exhibited that the perceived stress was higher in younger age groups, correlated with another study carried out in India.^67^, ^68^ A study conducted in the Philippines discovered that young generations are dealt with higher psychological aftermath from the ongoing pandemic than men, respectively.^39^ Interchangeably, the studies conducted before the COVID‐19 pandemic observed that stress scores tend to decrease with age.^33^, ^69^, ^70^ However, this trend was not detected in French studies.^71^ Moreover, the number of quarantine days also seems to increase the perceived stress as et al has conducted that the longer the forced isolation continues, the more destructive the mental health consequences are.^72^

The value of a proper resilience was observed through this study, and the need to maintain a good resilience was highlighted. In order to achieve this goal, we must first identify each factor that affects resilience in each group of people. One way is to integrate resilience enhancement programs into each occupation as a part of their staff support strategy system.^73^ Creating a safe work environment, manageable workloads, ensuring each individual feels valued, proper managing of the expectation by the leaders are some of the examples of how resilience can be enhanced in organizations. Additionally, individuals' strategies that can help maintaining a good resilience are improving adaptive skills, which are through problem solving, abstinence from avoidance, and seeking help.^74^

Iran has seen a dramatic increase in the number of people and deaths affected, the greatest in Asia (outside China). The Iranian outbreak was mainly due to a lack of initial government responses, limited public awareness of contagion risk, and a lack of compulsory self‐quarantine. Public perceptions are a key issue underlying Iran's high death rate. Along with the pandemic, various factors seem to influence the mental health of the Iranian people. The firm sanctions against Iran can be named among these. While sanctions against Iran have been in place for the past 40 years since the Islamic Revolution and have covered almost all sectors, such as insurance, banking, oil, trade, and transportation,^75^ the pandemic of COVID‐19 in Iran coincides with the ever‐increasing political‐induced sanctions against the country and during the national economic crisis, in which the price of medicine has risen sharply. This has created a dramatic social concern that has driven people to buy and stockpile medical supplies unnecessarily, resulting in shortages in other regions. Also, all religious services, including masses and Friday prayers, and religious congregations were closed, along with colleges and schools, entertainment centers, cinemas, theatres, and sporting activities and gyms; car and real estate transactions decreased; and hotels and accommodation centers received virtually zero guests.^76^ This causes the public to be deprived of entertainment centers, as well as affecting the worship patterns of the religious community, as Iran is among the religious countries in the Middle East, which we cannot ignore the effects on the mental health of individuals due to these factors. Jahanshahi et al say that adults in Iran experience more distress than adults in China, with different factors predicting the degree of distress.^77^ Our study was during the early days of the outbreak; therefore, it was still difficult to determine the effect of government and people's adopted policies on the mental health status of populations, but it cannot be denied that particular attention should be paid to safeguarding the populations' mental health alongside their physical health to prevent long‐lasting effects.

Mental health is considered to have one the most influential roles on general health of each individual, and the COVID‐19 brought a new wave of burden on mental health in each community. Through rapid advances in technology era and amid an outbreak, the substantial need for the internet rises each day and has empowered the delivery of such psychotherapy interventions via the internet. Numerous hospitals have started to provide psychotherapy to patients via video conferencing platforms. Our recommendation is that it is beneficial that government and health authorities take the role of telepsychiatry and referral, in case the need arises, more seriously and consider insurance coverage in order to provide consultation and initiate psychological intervention, such as mindfulness‐based therapy (MBT) and cognitive behavioral therapy (CBT), to lessen the rate of psychological issues and suicide. Behavioral therapy could teach relaxation techniques to overcome anxiety and prevent depression.^78^ Soh et al have conducted a meta‐analysis on the efficacy of digital CBT for insomnia and conclusively, provides strong support for the efficacy of digital CBT in treating insomnia, and can have a revolutionary role of changing the mode of implementing CBT so it can benefit patients worldwide.^79^ All in all, CBT has proven to be useful and effective for both medical and psychiatric conditions. Although implementing an internet‐based program costs a lot, some applications like Moodle, an open‐source learning platform, can be used to deliver such therapies in a more cost‐effective way.^80^

This pandemic has offered priceless lessons in terms of global responses, including, having better medical technology and workforce allocation while not disregarding the impact of psychology on individuals and society during and after the pandemic that is usually the restrictive aspect for the nation to deal with the crisis. By solidification of psychological defenses, the nations can endure fighting this long‐drawn combat and secure a successful future. Furthermore, health care professionals should adapt to deliver telepsychiatry services more. Lastly, vaccination plays a vital role in controlling the spread of COVID‐19. As of late September, almost 16% of the Iran's population are vaccinated, and as the more population gets vaccinated, the disease burden on physical and mental health of the people will decline.^81^, ^82^ A novel system should be created to identify people who are more at risk of mental health status due to their type of occupation and how the pandemic can influence their income and in solution, and a more organized insurance system should be arranged to help cover the lost income.

A longitudinal study conducted by Wang et al in china assessed the general population mental health during the initial outbreak and 4 weeks into the epidemic's peak. During their initial assessment, they observed moderate to severe stress, anxiety, and depression that did not have a significant longitudinal change, and high level of confidence in doctors, low risk of COVID‐19 contraction, and satisfaction with the health information were recognized as protective factors for mental health.^83^ As the present study was conducted at the time of the first wave of COVID‐19 pandemic in Iran, further longitudinal measurements need to be carried out in that order, which is one of the limitations this article has faced. The COVID‐19 pandemic was found to cause hemodynamic changes in the brain and impairment in olfactory function.^84^, ^85^ This study mainly used self‐reported questionnaires to measure psychiatric symptoms and did not make clinical diagnosis. The gold standard for establishing psychiatric diagnosis involved structured clinical interview and functional neuroimaging.^86^, ^87^, ^88^

Also, there were no studies regarding the general population's resilience status before the pandemic for comparison. There would have been more distinguishable results have we separated different occupations due to the difference of exposure to stress. Also, income level was not quantified and was assessed based on the participants' perception of their salary since we aimed to evaluate the participants' interpretation regarding this issue. Also, our population was an overrepresentative of high educational level individuals and also females. Moreover, as the process of vaccination is still in its early ages in Iran, despite primary studies discussing prioritizing strategies, no study has yet conducted the population's attitude toward vaccination; it is recommended that a cross‐sectional study be conducted on normal population and comparing their willingness to receive vaccine to those with mental health issues.^89^ Lastly, the possibility of occurring Bias is likely as the study was performed self‐reported.

## 5 CONCLUSION

The occurring COVID‐19 pandemic could be the culprit of psychological distress, anxiety, and depression of large population quantities. The overwhelming number of people infected each day cause a consequential burden on medical staff and psychologist. Utilizing PSS‐14, DASS‐21, and CD‐RISC exhibited slightly higher than normal perceived stress in the investigated population, notwithstanding a good resilience among our study group. The male population had a higher resilience than the female, and resilience was discovered higher in those with higher incomes.

In conclusion, this study implicated the importance of mental health and the groups who are in greater danger. These findings call for a more focused targeted interventions for women and those with low‐income salary. As most of the studies are conducted online, it is crucial to address this message to keep the underprivileged and remote rural people in mind and conduct future studies on heterogeneous community.

## FUNDING

None declared.

## CONFLICT OF INTEREST

The authors declare no conflicts of interest.

## AUTHORS' CONTRIBUTIONS

Formal analysis: Narges Ghamari.

Methodology: Seyedeh Yasamin Parvar.

Supervision: Narges Ghamari and Reza Shahriarirad.

Validation: Reza Shahriarirad.

Writing—original draft: Seyedeh Yasamin Parvar and Fatemehsadat Pezeshkian.

All authors read and approved the final version of the manuscript.

Dr Narges Ghamari had full access to all of the data in this study and takes complete responsibility for the integrity of the data and the accuracy of the data analysis.

## TRANSPARENCY STATEMENT

The Dr Narges Ghamari affirms that this manuscript is an honest, accurate, and transparent account of the study being reported and that no important aspects of the study have been omitted.

## CONSENT FOR PUBLICATION

The purpose of this research was completely explained to the participants through the cover page of the questionnaire, and they were assured that their information would be kept confidential by the researcher. Informed consent from the participants was acquired as they agreed to participate in the study by reviewing the questionnaire's cover page and clicking on the provided link. Furthermore, for participants younger than 18 years of age, the participant was asked for the consent of the parent or guardian.

## ETHICS STATEMENT

The Medical Ethics Committee of the university approved the present study according to the declaration of Helsinki.

## Data Availability

The data that support the findings of the present study are available on request from the corresponding author. However, they are not publicly available due to privacy and ethical restrictions.
